# Intimidation, harassment, and discrimination during family medicine residency training: a mixed methods study

**DOI:** 10.1186/s12909-021-02623-w

**Published:** 2021-03-20

**Authors:** Olga Szafran, Wayne Woloschuk, Jacqueline M. I. Torti, Maria F. Palacios Mackay

**Affiliations:** 1 Department of Family Medicine, University of Alberta, 6-10 University Terrace, Edmonton, Alberta T6G 2T4 Canada; 2 Cumming School of Medicine, University of Calgary, Room 701B, Health Sciences Centre, 3330 Hospital Drive NW, Calgary, Alberta T2N 4N1 Canada; 3 Centre for Education Research & Innovation, Schulich School of Medicine & Dentistry, Western University, Suite 100, Medical Sciences Building, London, Ontario N6G 2V4 Canada; 4 Interdisciplinary Centre for Education Innovation (CIED) Santo Tomás, Universidad Santo Tomás, Arturo Prat 866, Piso 5, CP 4100000 Concepción, Chile; 5 Department of Family Medicine, University of Calgary, Calgary, Alberta Canada

**Keywords:** Graduate medical education, Residency, Family practice, Harassment, Discrimination, Mistreatment

## Abstract

**Background:**

The importance of wellbeing of family medicine residents is recognized in accreditation requirements which call for a supportive and respectful learning environment; however, concerns exist about learner mistreatment in the medical environment. The purpose of this study was to to describe family medicine graduates’ perceived experience with intimidation, harassment and discrimination (IHD) during residency training.

**Methods:**

A mixed-methods study was conducted on a cohort of family medicine graduates who completed residency training during 2006–2011. Phase 1, the quantitative component, consisted of a retrospective survey of 651 graduates. Phase 2, the qualitative component, was comprised of 11 qualitative interviews. Both the survey and the interviews addressed graduates’ experience with IHD with respect to frequency and type, setting, perpetrator, perceived basis for IHD, and the effect of the IHD.

**Results:**

The response rate to the survey was 47.2%, with 44.7% of respondents indicating that they experienced some form of mistreatment/IHD during residency training, and 69.9% noting that it occurred more than once. The primary sources of IHD were specialist physicians (75.7%), hospital nurses (47.8%), and family physicians (33.8%). Inappropriate verbal comments were the most frequent type of IHD (86.8%). Graduates perceived the basis of the IHD to be abuse of power (69.1%), personality conflict (36.8%), and family medicine as a career choice (30.1%), which interview participants also described. A significantly greater proportion IMGs than CMGs perceived the basis of IHD to be culture/ethnicity (47.2% vs 10.5%, respectively). The vast majority (77.3%) of graduates reported that the IHD experience had a negative effect on them, consisting of decreased self-esteem and confidence, increased anxiety, and sleep problems. As trainees, they felt angry, threatened, demoralized, discouraged, manipulated, and powerless. Some developed depression or burnout, took medication, or underwent counselling.

**Conclusions:**

IHD continued to be prevalent during family medicine residency training, with it occurring most frequently in the hospital setting and specialty rotations. Educational institutions must work with hospital administrators to address issues of mistreatment in the workplace. Residency training programs and the medical establishment need to be cognizant that the effects of IHD are far-reaching and must continuously work to eradicate it.

## Background

The wellbeing of family medicine residents is vital as they function in multiple roles as learners, teachers and providers of patient care within the clinical setting. These roles require them to interact daily with numerous individuals and subject them to many stressors and pressures, that in turn affect their wellbeing and how they care for patients. Adverse stressors can take the form of intimidation, harassment and/or discrimination (IHD). The 2018 National Resident Survey in Canada reported that 78.2% of all residents and 69.5% of family medicine residents experienced at least one form of intimidation or harassment in the previous year [1]. This is consistent with a review of the published literature that found that between 45 to 95% of residents experience mistreatment or IHD at least once during residency training [2].

The most prevalent types of unwanted behaviors reported by residents include inappropriate verbal comments [1–5] and work as punishment [1, 4]. The dominant sources of IHD have been staff physicians, nurses and other health care providers [1, 2, 4–6], other residents [1, 4], and patients [1, 4–6]. Among residents, gender has been identified as the perceived basis of IHD [4, 7–11], with more females experiencing gender discrimination. Culture and ethnicity have also been identified as the basis of IHD [1–4], with more international medical graduates (IMGs) identifying culture/ethnicity and language as the perceived basis of IHD. Mistreatment and IHD have been reported to have a negative effect on residents including emotional impact [2, 5, 8] and burnout [12].

Studies on medical students’ experiences with mistreatment are more abundant in the literature than those on resident mistreatment. There are few mixed-methods and intervention studies aimed at reducing or eradicating IHD during residency training. In particular, there is a paucity of research on IHD experienced by family medicine residents and within the Canadian context, as well as IHD prevalence in different cohorts of residents over time. We undertook this study to build upon our previous research on IHD in a 2001–2005 cohort of family medicine graduates [4], to enable comparison of IHD prevalence over time, and to contribute to the Canadian literature. The purpose of this study was to to describe the 2006–2011 cohort of family medicine graduates’ perceived experience with IHD during residency training.

## Methods

An explanatory, sequential, mixed-methods design was employed which involved collecting quantitative and qualitative data sequentially in two phases. Phase 1, the quantitative component, consisted of a retrospective survey. Phase 2, the qualitative component, was comprised of qualitative interviews that were used to gain a deeper understanding of the survey findings from Phase 1. The study was approved by the Health Research Ethics Board (Health Panel), University of Alberta and the Conjoint Health Research Ethics Board, University of Calgary. All methods were carried out in accordance with relevant guidelines and regulations.

### Phase 1: retrospective survey

A retrospective survey was conducted of family medicine graduates (*n* = 651) who completed the residency programs at the University of Alberta or the University of Calgary during 2006–2011, inclusive. Each university mailed the survey to its graduates using a modified Dillman method [13]. Graduates’ contact information was obtained from the Alberta Medical Directory or the Canadian Medical Directory. The survey was conducted from July to December 2014.

The survey was based on previous questionnaires used to survey the 1985–95, 1996–2000, and 2001–2005 cohorts of Alberta family medicine graduates. In its entirety, the survey addressed several major areas: (a) medical education; (b) career history (clinical and non-clinical activities; current practice, practice location); (c) IHD; (d) well-being; (e) program evaluation; (f) perceptions about family medicine; and (g) demographics. This analysis of IHD is a subset of the larger study. The questions related to IHD addressed the frequency, type and source of IHD, perceived basis for the IHD, awareness of the process to address IHD issues within the residency program, and the effect the IHD had on the graduate. While IHD was not defined in the survey, in general terms intimidation, harassment, discrimination, and mistreatment were intended to refer to remarks, actions, or behaviours that were perceived to be unwanted, hurtful, upsetting, or coercive. Consent was implied by the return of a completed questionnaire.

### Phase 2: qualitative interviews

The last question on the survey asked respondents if they were willing to be contacted to participate in an interview as part of Phase 2 of the study. Those who replied “yes” and also indicated on the survey that they experienced IHD were contacted to take part in the IHD interviews. Those who agreed to be interviewed provided written informed consent and were interviewed via telephone between November 2015 and February 2016. The IHD interview questions addressed: (a) participants’ perspectives on the meanings of IHD; (b) their experience with IHD with respect to frequency and type, setting, perpetrator, perceived basis for IHD, and whether they reported it; (c) the perceived effect the IHD had on them; and (d) their awareness of others experiencing IHD during the residency program (Table 1).

**Table 1:** Interview Questions

1. What does intimidation mean to you?	
2. What does harassment mean to you?	
3. What does discrimination mean to you?	
4. What was your experience with intimidation, harassment, and/or discrimination (IHD) during the family medicine residency program?	
a. In what form did you experience IHD?	
b. In what setting(s) did you experience IHD?	
c. Who was the offender/perpetrator?	
d. What do you believe to be the basis/reason for the IHD?	
e. How often did you experience IHD?	
f. Did you report it?	
4. What effect has the IHD experience had on you?	
5. Were you aware of other family medicine residents experiencing IHD during the same time that you went through the program? Elaborate.	
6. What are your thoughts about your own or others’ experiences with IHD during family medicine residency training?	

Interviews were conducted by one of the authors (JT) who was trained in qualitative interviewing skills. At the time of the study, JT was a graduate student and did not have any prior relationship with the participants. The interviews were audio-recorded and transcribed verbatim by a professional transcription service. The transcripts were reviewed against the recorded interviews by one of the authors (JT) who edited the transcripts, as needed, to ensure data quality.

### Data analysis

Survey data were entered into SPSS 24 for Windows and analyzed using descriptive statistics, chi-square, and Fisher’s Exact Test, as appropriate. An alpha level of 0.05 was used to test for statistical significance.

Interview data were analyzed descriptively using qualitative content analysis [14]. Initially, three of the authors (MPM, OS, WW) independently read, reviewed and coded each transcript prior to group meetings during which time group discussions were held to reach consensus on agreed themes. Subsequently, the fourth author (JT) who conducted the interviews, read and coded each transcript at a later time as a form of peer-review and to provide feedback. MPM, OS and WW analyzed the transcripts to identify patterns within the data, while JT was able to confirm the findings based on interviewing the participants. This process took considerable time and contributed to improving the trustworthiness of the study findings.

## Results

### Survey Respondents & Interview Participants

A total of 307/651 (47.2%) graduates responded to the overall survey, of whom 304 responded to the IHD question. There was no statistically significant difference in demographic characteristics between the IHD group and all the respondents (Table 2). Overall, 44.7% (136/304) of respondents indicated that they experienced some form of mistreatment/IHD during residency training.

**Table 2:** Characteristics of Survey Respondents

Characteristics	Number (%)	
	All Respondents ***n*** = 307	Perceived IHD ***n*** = 136
**Gender**		
Male	116 (37.8)	39 (28.7)
Female	188 (61.2)	97 (71.3)
Transgendered	1 (0.3)	0 (0.0)
Not Recorded	2 (0.07)	0 (0.0)
Age		
≤ 34 years	105 (34.2)	41 (30.1)
35–44 years	138 (45.0)	60 (44.1)
≥ 45 years	52 (16.9)	30 (22.1)
Not recorded	12 (3.9)	5 (3.7)
**Medical School Graduates**		
CMG	236 (76.9)	98 (72.1)
IMG	69 (22.5)	37 (27.2)
Not Recorded	2 (0.7)	1 (0.7)

A total of 54 respondents who reported some form of IHD on the survey also expressed interest in being contacted to take part in an interview. However, upon follow up, just 11 agreed to be interviewed. The interviews ranged from 14.35 to 39.5 (mean = 22.8) minutes in duration. The characteristics of interview participants were: 3 males and 8 females; 7 CMGs and 4 IMGs; 10 University of Alberta and 1 University of Calgary graduates.

### Meaning of IHD

A key element to the interviews was to ask participants about their perceptions of the meaning of IHD. *Intimidation* was described as behavior that makes someone feel threatened, uncomfortable or bullied. *Harassment* was considered to be more severe than intimidation and to consist of active behaviors that make someone feel victimized. Participants associated intimidation and harassment with power imbalance and authority*. Discrimination* was noted to be unfair treatment, bias, stereotype or unjustified judgement based on some group characteristic (e.g. race, gender, religion, age, etc.). Discrimination was thought to make some people feel less valuable because of certain characteristics. The described meanings of IHD were consistent with our presumed meaning.

### Frequency of IHD

Thirty-seven (27.2%) survey respondents indicated that they experienced IHD only once and 95 (69.9%) indicated that it occurred more than once. Interview participants used words like *“a couple of times,” “two times,” “only on a couple of rotations,”* “*every time we had contact,” and “multiple times”* to describe the frequency of IHD occurrences that happened more than once.

### Source of perceived IHD

The primary sources of IHD were specialist physicians (75.7%), hospital nurses (47.8%), family physicians (33.8%), patients (26.5%), and specialty residents (24.3%) (Table 3). Interview participants confirmed that IHD occurred primarily in the hospital setting and on specialty rotations and the primary sources were specialty physicians, specialty residents and hospital nurses.

**Table 3:** Overall Intimidation, Harassment & Discrimination

	Number (%) *n* = 136
**Type of IHD**	
Inappropriate verbal comments	118 (86.8)
Work as punishment	22 (16.2)
Recrimination for reporting	16 (11.8)
Privileges/opportunities taken away	14 (10.3)
Inappropriate/unwanted physical contact	5 (3.7)
Sexual harassment	5 (3.7)
Other	21 (15.4)
**Source of IHD**	
Specialist physicians	103 (75.7)
Hospital nurses	65 (47.8)
Family physicians	46 (33.8)
Patients	36 (26.5)
Specialty residents	33 (24.3)
Family medicine residents	12 (8.8)
Program director	9 (6.6))
Support staff	7 (5.1)
Family medicine nurses	3 (2.2)
Other	5 (3.7)
**Perceived Basis for IHD**	
Abuse of power/power tripping	94 (69.1)
Personality conflict	50 (36.8)
Choice of family medicine specialty	41 (30.1)
Gender	28 (20.6)
Culture/ethnicity	28 (20.6)
Religion	8 (5.9)
Language	7 (5.1)
Sexual orientation	0 (0.0)
Other	14 (10.3)

### Type of perceived IHD

Of the family medicine graduates who reported IHD, survey findings revealed that inappropriate verbal comments (86.8%) were the most frequent type of IHD, followed by work as punishment (16.2%) (Table 3). Examples of work as punishment included being assigned a higher workload or being asked to work shifts that are less desirable or to dictate summaries on patients the resident had not seen. The ‘other’ category included belittling, blaming, disrespecting, having unreasonable expectations/demands, and laughing in the background. Interview participants elaborated on the inappropriate verbal comments describing perceived aggressive/excessive criticism, verbal berating and belittling, swearing, shaming, humiliating comments, shouting and yelling, and public embarrassment.

*“What’s wrong with you … you’re as dense as a brick wall … you’re not any help now, and just told me to get out basically.”* (Interview 9)

*“…he [staff surgeon] looked at it and told me that I had written too many words on the consult sheet and then he ripped it up and threw it in the garbage and told me that I had to go back and redo the consult, which had taken about an hour and a half, and keep in mind that I’ve been up for 26 hours, I’m exhausted,…and not only that, but I didn’t even have the consult information to utilize to redo from my notes because he’d thrown it away.”* (Interview 4)

*“I just remember her using words like ‘You’re not pulling your weight’ and then shouting, just yelling … yelling during deliveries, during assessments,…It was bullying.”* (Interview 10)

Participants also commented that on specialty rotations they felt that they experienced differential treatment compared to specialty residents. Derogatory insinuations were made by others that family medicine residents were *“somehow dumber than the rest”* (Interview 2). Graduates felt that, sometimes on specialty rotations, they were assigned duties and opportunities that were perceived to be less desirable (*“crappy stuff”* (Interview 2)) than those assigned to specialty residents, and were not considered for certain clinical experiences on the mistaken or uninformed assumption that certain opportunities were not essential for family practice.

*“I had asked about going to clinics and one of the preceptors said, ‘You belong in the hospital, you don’t need to be seen in the clinics because in family medicine we never see gynecology.’”* (Interview 3)

Participants noted that family medicine residents were excessively criticized and unfairly compared to specialty residents who were much further along in their residency training.

Graduates also described IHD in the form of perceived threats - threats of bad evaluations, threats of being reported.

*“There was a kind of a threat of a bad review or bad evaluation if you didn’t act in a certain way or cater to certain people.”* (Interview 1)

“*She [nurse] would page us at all hours…she would sort of make it known that she would let our staff know if we weren’t doing everything she wanted.”* (Interview 1)

Two interview participants described physical forms of IHD.

*“…when I was doing my training, one of the surgeons threw a trolley cart at a student, missed.”* (Interview 3)

*“…I had a close friend of mine…the staff person had thrown a pair of scissors at her because they were upset with something…it’s both dangerous and incredibly unprofessional.”* (Interview 4)

### Basis of perceived IHD

Survey findings revealed that the basis for the IHD was perceived to be an abuse of power or power tripping (69.1%), personality conflict (36.8%), and family medicine as a career choice (30.1%) (Table 3). Gender or culture/ethnicity was each noted to be the basis of IHD by 20.6% of respondents who reported IHD. The ‘other’ category included, but not limited to, the belief that family medicine residents were unqualified, jealousy, preceptor stress, and unrealistic expectations of the role of the resident as a learner and service provider.

Interview participants concurred that power tripping was the basis of the IHD and speculated that those with low self-esteem used IHD to boost or reinforce their authority and self-worth. This behavior was deemed by participants to be an abuse of power. Participants described hearing remarks related to hierarchy and power differences between the specialties and family medicine, with family medicine being at the bottom and perceived to have less value than the specialty disciplines. They also conveyed hearing subtle inferences that family medicine residents were not as smart as specialty residents. Study participants felt that their skills were frequently underestimated.

*“…this sort of underlying belief that only people who can’t get into a specialty get in family [medicine]. It just kills me.”* (Interview 2)

*“He was feeling sorry for me that I’m a family physician.”* (Interview 5)

Graduates also attributed the IHD to frustrations related to a heavy workload. They noted that the demands of clinical care, coupled with a lack of effective stress coping strategies (lack of good impulse control), triggered individuals to direct their anger out on someone else.

*“… people get frustrated and overworked and tired, and then it’s easy to say things or take things out on other people…”* (Interview 11)

*“…I think it occurs because in medicine it’s a stressful environment…just like other stressful situations, it brings out the best in people, but also sometimes brings out the worst in people.”* (Interview 11)

### Gender

A significantly greater proportion of females (51.9%) than males (33.9%) (*p* = 0.003) indicated experiencing IHD. There were no differences in the types or sources of perceived IHD by gender; however, a significantly greater proportion of females (26.6%) than males (7.9%) indicated that the perceived basis for IHD was gender (*p* = 0.02).

### Age

There was a trend in increasing prevalence of IHD (39.4, 43.5, 57.7%) with increasing age group (≤ 34 years, 35–44 years, ≥ 45 years), respectively (*p* = 0.04). With increasing age group, a significantly greater proportion of graduates identified specialty residents (15.4, 24.6, 47.6%; *p* = 0.009) as the sources of IHD. In addition, culture/ethnicity was perceived to be the basis of IHD with increasing age group (2.5, 25.9, 37.9%, p = < 0.001).

### CMG & IMG

No statistically significant difference in the prevalence of perceived IHD between CMGs (42.1%) and IMGs (53.6%) (*p* = 0.10) was observed. However, a significantly greater proportion of CMGs than IMGs indicated that they experienced IHD in the form of inappropriate verbal comments (92.7% vs 77.8%) and perceived the basis of IHD to be an abuse of power (78.1% vs 52.8%) or gender (28.4% vs 2.8%), respectively (Table 4). In contrast, relatively more IMGs than CMGs reported that the sources of perceived IHD were family medicine residents (29.6% vs 4.3%), program director (19.2% vs 4.3%), or support staff (15.4% vs 3.2%), respectively (Table 4). Also, more IMGs than CMGs perceived the basis of IHD to be culture/ethnicity (47.2% vs 10.5%) or language (13.9% vs 2.1%), respectively (Table 4).

**Table 4:** Type, Source & Perceived Basis of IHD by CMG and IMG

	CMG ***n*** = 98 (%)^*^	IMG ***n*** = 37 (%)^*^	***p*** value^**^
**Type of IHD**			
Inappropriate verbal comments	89 (92.7)	28 (77.8)	0.03
Work as punishment	17 (18.1)	5 (13.9)	0.79
Recrimination for reporting	11 (11.7)	5 (13.9)	0.77
Privileges/opportunities taken away	10 (10.6)	4 (11.1)	1.00
Inappropriate/unwanted physical contact	4 (4.3)	1 (2.8)	1.00
Sexual harassment	5 (5.4)	0 (0.0)	0.32
**Source of IHD**			
Specialist physicians	74 (77.1)	28 (87.5)	0.31
Hospital nurses	47 (50.0)	17 (58.6)	0.52
Family physicians	31 (32.6)	15 (50.0)	0.13
Specialty residents	21 (22.6)	11 (40.7)	0.08
Patients	30 (31.9)	6 (23.1)	0.47
Family medicine residents	4 (4.3)	8 (29.6)	0.001
Program director	4 (4.3)	5 (19.2)	0.02
Support staff	3 (3.2)	4 (15.4)	0.04
Family medicine nurses	3 (3.2)	0 (0.0)	1.00
**Perceived Basis for IHD**			
Abuse of power/power tripping	75 (78.1)	19 (52.8)	0.01
Personality conflict	38 (40.0)	12 (33.3)	0.55
Choice of family medicine specialty	31 (32.6)	9 (25.0)	0.52
Gender	27 (28.4)	1 (2.8)	0.001
Culture/ethnicity	10 (10.5)	17 (47.2)	< 0.001
Religion	3 (3.2)	4 (11.1)	0.09
Language	2 (2.1)	5 (13.9)	0.02
Sexual orientation	0 (0.0)	0 (0.0)	–

^*^Denominator for the calculation of percentages varies due to missing values

^**^ Fisher’s Exact test

### Effect of IHD

The vast majority (77.%) of graduates reported a negative effect of the IHD, with 18.9% indicating a very negative effect and 58.3% noting a somewhat negative effect. Written comments on the survey and interview data revealed that the IHD experience decreased their self-esteem and confidence, increased anxiety, and resulted in sleep issues. As trainees, they felt angry, threatened, demoralized, discouraged, manipulated, and powerless. Some expressed fearing the consequences of power differences between themselves and their perpetrators (bad evaluation, failing the rotation) and fear of retaliation. A few reported that they had developed depression or burnout and sought the services of mental health professionals. For some, the IHD had resulted in resentment toward the perpetrator and the perpetrator’s clinical specialty to the extent that it influenced their decision not to include particular types of clinical care (e.g. obstetrics) in their future practice. The IHD experience had decreased professional satisfaction and some had even thought of dropping out of the residency program. For some, the anxiety continued well into the future when dealing with the particular specialist years afterward.

*“I was shaking.”* (Interview 3)

*“…I could hardly wait for it [rotation] to be over, I didn’t care if I passed or failed at that point. I just wanted to get out of there, just finish it and just be done with it.”* (Interview 7)

*“…I could not sleep, I took medications as well…I went for counselling for months and months.”* (Interview 8)

*“…I said for a long time I would never want my children to do medicine.”* (Interview 3)

*“I still don’t refer to that hospital because of that doctor.”* (Interview 3)

Some residents reported either no effect (15.2%), a somewhat positive effect (6.8%), or a very positive effect (0.8%) of the IHD. A few participants described positive outcomes which included that the experience made them more confident in confronting inappropriate behavior, and made them more open-minded, observant, and motivated to change perceptions. A few of the participants did not take the IHD personally and did not report any long-lasting effects. Some indicated that they were motivated to change perceptions of family medicine through education.

*“…I felt sorry for him that he had to go to those lengths just to make himself look good...”* (Interview 7)

*“I think it made me more assertive and more confident.”* (Interview 10)

### Reporting IHD

Of those graduates who experienced IHD, 52.2% were aware of the process to report IHD within the program, but only 22.8% used the process. Most interview participants indicated that they told their family or friends about the IHD incident, and some told their family medicine advisor/preceptor or voiced their concerns through the Discipline Advisory Groups process (rotation review group), however, most did not file an official complaint. The reasons for not reporting the IHD included the belief that nothing would be done to change it anyway, fearing that the IHD would get worse, and fear of retaliation of a bad rotation evaluation. Some seemed to be complacent that IHD was endemic in the health care workplace.

*“…I don’t think it would have gone anywhere and I wouldn’t want the hassle. Cause those people are not going to change what they think.”* (Interview 2)

*“Was apprehensive to report because I thought the bullying was going to get worse and that people would gang up against you, and I was concerned about my evaluation”* (Interview 10)

*“I don’t think I would have used it to be honest, no. … One, it was an isolated incident and it wasn’t ongoing, so I just moved on … and forgot about it … Two, I think there is an awareness that things like this they’re not easily fixed. They’re sort of endemic within the system, everyone knows that …if it was something else that was much more dangerous or prolonged, perhaps.”* (Interview 9)

The few who had reported the IHD expressed appreciation for the support they received from the family medicine program in addressing the issue.

## Discussion

The study findings reveal that IHD remained prevalent during residency training during 2006–2011. The 44.7% of family medicine graduates who reported experiencing IHD in this study is identical to the 44.7% who reported IHD in the 2001–2005 cohort we studied earlier [4]. It is disconcerting that, despite increasing awareness to mitigate mistreatment, the prevalence remained unchanged over the two cohorts spanning 11 years. While the reason for this remains unclear, the findings are consistent with a longitudinal study of medical students which showed that despite proactive measures over 13 years to eradicate mistreatment, it persisted at consistent rates for three study periods [15]. Much of behavior is learned through role modeling, therefore, if physicians serve as role models for residents, and residents are role models for medical students [16], then this cascade effect may help to elucidate why the prevalence of IHD had not changed over time.

Consistent with other published studies [2, 4], mistreatment was noted to occur most often on clinical rotations within the hospital setting and the two main sources of IHD were specialty physicians and hospital nurses, individuals that residents interact with frequently. Our findings are consistent with other studies that report high levels of mistreatment on surgical [5, 17–21], obstetrics/gynecology [19, 21] and internal medicine [5, 21] rotations. It is possible that different norms as to what constitutes mistreatment may exist in family medicine versus other medical specialties. Studies have also found that medical students who were interested in a specialty career reported mistreatment more often than those interested in primary care [19, 21]. The acute care hospital setting is a high-stakes, stressful work environment with hierarchical power structures. There is a need to mitigate the stressors in such intense work situations and hospital administration must play a significant role in addressing issues of mistreatment in the workplace.

Consistent with the published literature [1, 2, 4, 19], inappropriate verbal comments were the most frequent types of IHD. While appropriately challenging residents on clinical content should not be taken to be intimidation or harassment, inappropriate verbal comments that occur in the context of teaching and in the presence of others, may be perceived to be humiliating [22]. Intimidating or humiliating teaching practices may be used as a means of exerting dominance. Learners have postulated that teachers use intimidation and humiliation in teaching because they do not possess effective teaching skills [23]. Professional development aimed at equipping teachers with a range of effective teaching skills may be one strategy to combat the prevalence of learner mistreatment.

Family medicine graduates in our study experienced differential treatment compared to specialty residents in the form of derogatory comments about the discipline of family medicine, being denied learning opportunities, or feeling that they would receive lower evaluations because they were in family medicine. These constitute part of the hidden curriculum [16] which, taught through implicit behaviors, undermines the formal curriculum and denigrates the discipline of family medicine.

Distinct differences between CMGs and IMGs appear to exist related to the basis of IHD. CMGs attributed IHD mainly to abuse of power or power tripping as a means to exert medical dominance of the specialty disciplines, whereas IMGs perceived the basis of IHD to be culture/ethnicity or language. IMGs have reported ethnicity as the perceived basis of mistreatment in a previous study [4]. Some IMGs may come from cultures where power is not challenged, therefore, would not have attributed the basis of IHD to an abuse of power in the work or education setting. Based on country of medical degree (not cultural/ethnic background), IMGs tend to be a minority group within Canadian residency programs. *Minority stress theory* suggests that difficult social situations produce stress for individuals of minority groups and such stress has a cumulative effect over time [24, 25]. If IMGs immigrated from countries in which they experienced discrimination or persecution, over time this may result in a pervasive state of feeling discriminated against and disenfranchised. As such, IHD experiences may have a more powerful effect on IMGs and on their psychological wellbeing which may account for significantly more IMGs than CMGs perceiving the basis of IHD to be culture/ethnicity or language. It is also possible that some graduates may have interpreted negative feedback as IHD and as a personal attack on their being and attributed it to ethnicity or language issues.

IHD has an immediate and a long-lasting impact on family medicine residents. Immediate negative effects include experiencing anger, increased anxiety, sleep disturbance, and decreased confidence which can affect clinical performance in the delivery of patient care. Some graduates have developed depression and mental health issues requiring treatment and/or counselling which can have long-lasting effects. IHD experiences also appear to influence career choice decisions and practice referral decisions. The negative effects reported in our study are similar to those reported by doctors who experienced rude and dismissive communication [26]. Residency training programs and the medical establishment need to be cognizant that the effects of IHD are far-reaching and must continuously work to eradicate it.

Our survey findings reveal that 52.2% of graduates knew about the process of reporting IHD within the program, but only 22.8% actually reported it. The interview data, however, indicate that most told someone else (e.g. family member, friend, another resident, advisor) about it. While there seems to be a psychological need to share the IHD experience with someone, most do not file an official complaint because they do not believe that anything will be done about it. Residents perceive that there exists a culture that does not seem to change and, unfortunately, our IHD prevalence data from the 2001–2005 and 2006–2011 cohorts of residents seem to support this. A shift in the medical culture to create an environment that is open to addressing issues of IHD without fear of retribution and that can lead to resolution is necessary. Residency programs should communicate to residents early in training that it is acceptable for residents to discuss issues of IHD and that support is available. A change in attitudes and efforts aimed at building effective relationships between family medicine and the specialties are warranted. Educational institutions must also work with hospital administration and staff to eradicate IHD. Some have reported success by creating a formal departmental committee consisting of both faculty and residents with a mandate to address intimidation [27].

While the main strength of our study is its mixed-methodology which enabled the corroboration of survey data with more descriptive interview data, the study has several limitations. The results reflect the 2006–2011 cohort of family medicine residents and may or may not apply to current residents’ experiences. The 47.2% survey response rate is comparable to mailed surveys [28], but is lower than the 62–72% we obtained from similar surveys we conducted on 1985–95, 1996–2000, and 2001–2005 cohorts of Alberta family medicine graduates. Survey fatigue may have contributed to the lower response rate in the 2006–2011 survey. The retrospective, cross-sectional nature of the survey meant that graduates recounted IHD experiences several years after it occurred, thereby introducing recall bias, which may have acted to underestimate the overall prevalence of IHD. In not wanting to influence respondents’ perceptions of IHD, we did not specifically define IHD in the survey; as such, respondents may have interpreted it differently. While an important aspect of the study was the individual’s perception of IHD, not necessarily the specific definition, interview data confirmed that there was agreement on the definition of IHD. Interviewees volunteered or self-selected to participate in the study, thereby, resulting in possible respondent bias. It is unknown what motivated participants to take part in the interviews. It is possible that those on whom IHD had the greatest effect or those who had an “axe to grind” were more inclined to take part. Also, some participants may have accentuated their perceptions of IHD as a way of conveying that more needs to be done to address issues of IHD during residency training. Interview participants were primarily from one medical school, which may not reflect the experiences of graduates from other medical schools.

Future studies are needed on IMGs’ perceived basis of IHD related to culture/ethnicity and language. Also, there is a need to disentangle issues related to negative feedback on performance and IHD. Studies to address the impact of teaching by humiliation are warranted.

## Conclusions

Unfortunately, IHD continued to be prevalent during the 2006–2011 family medicine residency period, with it occurring most often in high-stakes, stressful medical environments with hierarchical power structures. While some stress is expected in the clinical learning environment, IHD can have far-reaching consequences that threaten the personal and professional wellbeing of residents and affect patient care. Residency training programs and the medical establishment must continually work to eradicate IHD.

## Data Availability

The data used during the current study are available from the corresponding author on reasonable request.

## Abbreviations

CMGs: Canadian medical graduates
IHD: Intimidation, harassment, discrimination
IMGs: International medical graduates
